# Machine Learning-Based Classification of Abnormal Liver Tissues Using Relative Permittivity

**DOI:** 10.3390/s22249919

**Published:** 2022-12-16

**Authors:** Poulami Samaddar, Anup Kumar Mishra, Sunil Gaddam, Mansunderbir Singh, Vaishnavi K. Modi, Keerthy Gopalakrishnan, Rachel L. Bayer, Ivone Cristina Igreja Sa, Shalil Khanal, Petra Hirsova, Enis Kostallari, Shuvashis Dey, Dipankar Mitra, Sayan Roy, Shivaram P. Arunachalam

**Affiliations:** 1 Microwave Engineering and Imaging Laboratory (MEIL), Division of Gastroenterology & Hepatology, Department of Medicine, Mayo Clinic, Rochester, MN 55905, USA; 2 GIH Artificial Intelligence Laboratory (GAIL), Division of Gastroenterology and Hepatology, Department of Medicine, Mayo Clinic, Rochester, MN 55905, USA; 3 Department of Radiology, Mayo Clinic, Rochester, MN 55905, USA; 4 Gastroenterology Research, Division of Gastroenterology and Hepatology, Mayo Clinic, Rochester, MN 55905, USA; 5 Department of Biological and Medical Sciences, Faculty of Pharmacy in Hradec Králové, Charles University, Heyrovskeho 1203, 500 05 Hradec Kralove, Czech Republic; 6 Department of Electrical and Computer Engineering, North Dakota State University, Fargo, ND 58105, USA; 7 Department of Computer Science, University of Wisconsin, La Crosse, WI 54601, USA; 8 Department of Electrical Engineering and Computer Science, South Dakota Mines, Rapid City, SD 57701, USA; 9 Department of Medicine, Mayo Clinic, Rochester, MN 55905, USA

**Keywords:** relative permittivity, dielectric spectroscopy, machine learning, microwave, non-alcoholic steatohepatitis, fibrosis

## Abstract

The search for non-invasive, fast, and low-cost diagnostic tools has gained significant traction among many researchers worldwide. Dielectric properties calculated from microwave signals offer unique insights into biological tissue. Material properties, such as relative permittivity ($\varepsilon_{r}$) and conductivity (σ), can vary significantly between healthy and unhealthy tissue types at a given frequency. Understanding this difference in properties is key for identifying the disease state. The frequency-dependent nature of the dielectric measurements results in large datasets, which can be postprocessed using artificial intelligence (AI) methods. In this work, the dielectric properties of liver tissues in three mouse models of liver disease are characterized using dielectric spectroscopy. The measurements are grouped into four categories based on the diets or disease state of the mice, i.e., healthy mice, mice with non-alcoholic steatohepatitis (NASH) induced by choline-deficient high-fat diet, mice with NASH induced by western diet, and mice with liver fibrosis. Multi-class classification machine learning (ML) models are then explored to differentiate the liver tissue groups based on dielectric measurements. The results show that the support vector machine (SVM) model was able to differentiate the tissue groups with an accuracy up to 90%. This technology pipeline, thus, shows great potential for developing the next generation non-invasive diagnostic tools.

## 1. Introduction

The capacitive behavior of any material, such as biological tissues, when exposed to a time-varying electric or magnetic field, is expressed as its ‘Dielectric Property’. Such time-varying electromagnetic fields create a combination of conduction and displacement currents through the tissue. As both currents appear to be a function of the frequency, the dielectric property is also a function of the frequency. The interpretation of the observed currents over the frequency spectrum offers information about the tissue’s characteristic structure and behavior. Now, data collected from such a material property can be used to train machine learning models to conveniently differentiate various tissues. Over the last few decades, many studies have been published showing the dielectric signatures of various biological tissues. Hence, such characterization has the potential to revolutionize various diagnostic and therapeutic modalities that are currently used in evidence-based medicine.

Gabriel et al. made the first attempt in 1996 to create a database of dielectric characteristics of various tissues. They obtained data from 40 different tissue types, at frequencies ranging from 10 Hz to 20 GHz [1,2,3]. Since then, many researchers have used this dielectric property database to showcase potential diagnostic solutions. It has been used to compare bone mineral density to dielectric properties, with the intent of developing a novel, harmless diagnostic test in place of currently used DXA (dual-energy X-ray absorptiometry), which exposes the patient to high doses of ionizing radiation [4]. It has ventured into the field of oncology, as many studies use the dielectric properties to differentiate normal (benign) and malignant breast tissue, primarily for breast cancer detection and treatment techniques [5,6,7,8]. It can also differentiate normal liver tissues from cirrhotic and malignant tissues [9]. Moreover, research has shown that dielectric spectroscopy can be used for blood glucose monitoring and continuous monitoring of hematocrit, which can be further used for renal dialysis [10,11]. Interestingly, it is also being devised to differentiate distinct types of renal stones (uric acid, struvite, calcium oxalate, calcium phosphate, etc.) [12], thus reducing the cost, turnaround time, difficulty of application while improving the efficacy, and sensitivity of currently available methods. Recently, some research works have also been published for blood flow velocity mapping, gastrointestinal tissue classification, and arterial waveform sensing based on dielectric spectroscopy techniques [13,14,15]. We believe that these findings can lead to the development of non-invasive microwave-based dielectric spectroscopic instruments for cancer detection in potentially every organ system of the body.

Despite the numerous attempts, this technique is still plagued by various issues, and one of the significant challenges going forward is the sheer number of diseases and tissue characteristics that need to be studied. Additionally, identifying useful data points from the enormous dataset to effectively train the machine learning models is another significant challenge. Active research is being performed to study the different disease models, collect characteristic data, and apply machine learning models. H. Rahmani et al. documented a wound monitoring platform, where they used supervised learning classification tools (i.e., support vector machine (SVM), Gaussian process, neural net, and naïve Bayes) to classify the permittivity of normal and wounded skin, based on their electrical properties (loss tangent). Although they measured three types of wounds (scratch, Ultraviolet (UV,) and punch), the classification models were run among only two classes [16]. A. Helwan presented a technique to classify breast tissues using machine learning [17]. Using a radial basis function-based neural network (RBFN), they classified the electric impedance of six types of breast tissues (carcinoma, fibro-adenoma, mastopathy, glandular, connective, and adipose tissue), with an average 87% recognition rate for test samples. T. Yilmaz et al. documented a project where they classified the dielectric properties of healthy and hepatic malignancies rat liver tissue with SVM classification algorithm with 99% accuracy. Despite having three types of tissues (malign, benign, and cirrhotic), only binary classification was performed between benign and malign types of tissues [18].

Currently, several techniques are well-established and commercially available for measuring the dielectric properties of biological tissues, such as the transmission line, cavity, tetrapolar (or multi-electrode) probe, and open-ended coaxial probe techniques [19]. The coaxial probe technique is the most popular, and it is non-destructive, broadband, and relatively straightforward [20,21]. This work utilizes the coaxial probe technique to measure the dielectric properties of the liver tissue samples gathered from healthy and diseased mice. Measurements were collected at a frequency spectrum from 0.5 GHz to 20 GHz. Dielectric constant or relative permittivity of an electrically lossy material, such as biological tissues, is a complex number, where the real part signifies the amount of electrical energy stored in a tissue, and the imaginary part denotes the energy loss. Gathering measurements throughout the frequency range is necessary, as the dielectric properties of the normal and abnormal tissue types are frequency dependent.

Recently, artificial intelligence (AI) has found many applications in the medical field. Different AI assisted diagnosis and treatment plans are already approved by regulatory agencies for clinical applications. The efficacy of dielectric property-based diagnostic tools can be greatly amplified with AI in studying diseased tissues. Previous studies have focused on using machine learning models to classify two tissue types with binary classification, namely healthy or diseased groups, using dielectric properties. In this work, we aim to use multi-classification models to differentiate between healthy liver tissues, different types of non-alcoholic steatohepatitis (NASH), and fibrosis liver diseases.

## 2. Materials and Methods

Initially, a commercially available, open-ended coaxial slim form probe and a vector network analyzer (VNA) were used on the tissues under test to measure the reflection coefficients (S11) from 500 MHz to 20 GHz. Relative permittivity ($\varepsilon_{r}$) values over that frequency range were then extracted from the measured S11 parameter for classification purposes. Mice liver tissues were measured and sent to the pathology department for histopathological analysis.

### 2.1. Experimental Procedures

#### 2.1.1. Animal Experiments

All animal experiments were performed by Mayo Clinic, Rochester, USA, and approved by the Institutional Animal Care and Use Committee. C57B1/6J mice were purchased from Jackson Laboratory (Bar Harbor, ME, USA). All mice were kept under specific pathogen-free conditions in a temperature-controlled animal facility at Mayo Clinic. All mice received humane care with free access to food, water, and a 12-h light/dark cycle.

Out of the total 72 mice, 25 mice were named as ‘healthy’. A total of 5 of them were fed for 24 weeks, with 10 of them were fed for 6 weeks, a standard chow diet before harvesting liver samples. The last 10 mice were injected with olive oil for 6 weeks. These are grouped together as ‘healthy’ because no diseases were induced among them, and dielectric properties measured from these tissue samples showed similar characteristics. 

Non-alcoholic steatohepatitis (NASH) model 1 was induced in 27 mice by a choline-deficient high-fat diet (CD-HFD), composed of high fat (60% calories) with 0.1% methionine and no added choline (#A06071302i, Research Diets, New Brunswick, NJ, USA), and mice received this diet for six weeks. For this study, these were named the ‘Diseased 1’ group.

NASH model 2 used a diet termed FFC (high fat, fructose, and cholesterol), composed of high fat (40% calories) and high cholesterol (0.2%) (#AIN-76A, TestDiet, St. Louis, MO, USA), with additional fructose (23.1 g/L) and glucose (18.9 g/L) added to drinking water. This third group was named ‘Diseased 2’ and contained 9 mice.

The last group consisted of 11 mice that received intraperitoneal injection of carbon tetrachloride (CCl4, 1 μL/g of body weight, #319961, Sigma-Aldrich, St. Louis, MO, USA) twice a week for six weeks, as described in [22,23,24], to induce liver fibrosis and were named ‘Diseased 3’.

Mice were sacrificed at the end of the feeding period for the healthy, ‘diseased 1’, and ‘diseased 2’ groups, as well as 48 h after the last infection for ‘diseased 3’ group. Liver tissue was collected at room temperature (20–22 °C), and ex vivo dielectric properties were measured within 3–30 min after sacrifice. A study by L. Farrugiaa et al. showed that ex vivo measurements kept well-hydrated and at body temperature can be a good approximation to in vivo measurements [25,26]. Hence, the samples were not immersed in any preserving liquid before or during measurements, and measurements are performed as soon as possible to closely represent the in vivo characteristics of the tissue. Each sample was measured multiple times at multiple locations of both sides of the sample for better representation. Table 1 shows the total count of the samples and measurements by tissue type.

#### 2.1.2. Measurement Setup

Liver samples were measured with the slim-form probe, manufactured by Keysight [27]. The instrument was calibrated from 500 MHz to 20 GHz before measurements by applying the OSL (open, short, and load) technique. To achieve an open condition, the probe was kept in the air. The short was measured by connecting the probe with a shorting block. Deionized water at room temperature (25 °C) was used as a load. After the calibration process, three standard liquids (methanol, ethanol, and isopropyl alcohol) were measured to validate the process. The results obtained after calibration were compared with reference permittivity of the liquids gathered from the literature. If needed, the calibration process was repeated.

After successful calibration, the samples were measured. The sample on a plastic dish was placed on a mechanical stage that could be moved vertically. This flexibility allowed us to place the sample evenly in contact with the probe end before starting the measurement. The pressure added by the probe on the measured tissue was estimated using a scale. The weight scale reading was kept at 1–2 gm for all measurements to minimize measurement error. To minimize the reflection from the metallic stand and weight scale, the plastic dish containing the tissue sample was placed on a non-conducting 3 cm thick styrofoam block.

**Figure 1:**
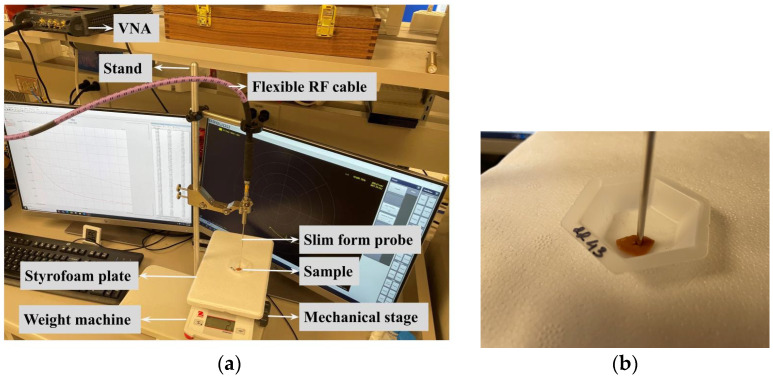
(**a**) Laboratory setup for the dielectric spectroscopy measurements using opened coaxial probe method at room temperature and (**b**) probe inserted into the sample under investigation.

Approximately 5–10 mm long, 3–8 mm wide, and 2–5 mm thick liver samples were measured during the experiments. Dielectric spectroscopy measurements were performed using an open-ended coaxial slim-form probe (N1501A Dielectric Probe Kit, Keysight Technologies, Santa Rosa, CA, USA). The probe was connected to a vector network analyzer (VNA) (P9374A, Keysight Technologies) with a flexible RF cable with SMA connectors. Figure 1a shows the laboratory setup used for dielectric spectroscopy measurement. A personal computer with VNA software (USB Network Analyzer, release 07.0, MathWorks. Inc, Natick, MA, USA) was connected to the VNA, which was used to control and save the reflection coefficient (S11) measurements. Another software, Keysight Materials Measurement Suite (version 18, Keysight Technologies, Santa Rosa, CA, USA), was used to convert the S11 parameter to the equivalent complex permittivity.

**Figure 2:**
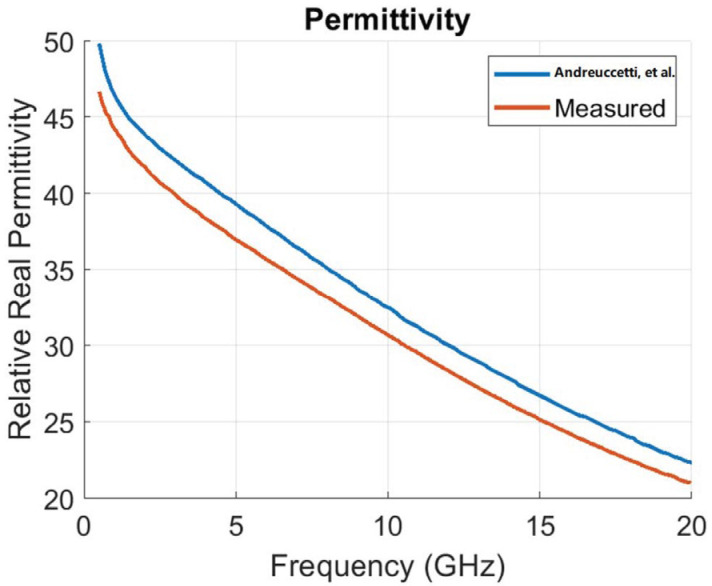
Comparison of reference [30] and measured real permittivity.

According to the instrument specification, the sample should be at least 5 mm around the probe to measure adequacy [27]. M. Cavagnaro et al. presented extensive work regarding this issue [28]. According to their findings, during measurement of a sample with high water content, such as liver, the probe should be inserted for at least 3 mm into the sample, and at least 3 mm tissue should be present between the tip of the probe and the bottom of the surface to maintain 5% accuracy threshold. Sometimes when dealing with biological tissues, these conditions are hard to follow. Some recent works were also published to understand the effect of probe position and variable thickness of tissue samples [20,21]. Another work by Porter et al. suggested that the tissue in contact with the probe has a greater impact on the measured dielectric properties than deeper tissues [29]. Considering all these studies, during our experiments, in most of the cases, we made an insertion in the liver sample, so that the probe can be inserted inside the sample, as shown in Figure 1b. Though the liver tissue samples were not always unform in thickness, the measurements were performed at multiple locations (5 mm thick location, as well as 2 mm thick location) and both inside and outside of the insertion. A standard protocol is always followed during all the measurements, so that all the measured data can be comparable. A comparative plot was also performed with measured (healthy liver tissue) average real permittivity and real permittivity (for liver tissues), as published in the literature [30]. Figure 2 shows our measured data closely followed the published data.

### 2.2. Data

The dielectric property or complex permittivity (ϵ(ω)) is a complex term dependent on angular frequency (ω), as shown in Equation (1). The real part ($\epsilon_{r}^{\prime }\left(\omega \right)$)is called relative permittivity [1], and it is the measure of the energy stored in the tissue from an external electric field. The imaginary part ($\epsilon_{r}^{\prime \prime }\left(\omega \right)$) is the dielectric loss factor, which suggests the dissipative nature of the tissue by energy absorption and partial heat conversion [13,20,21].

(1) $$\epsilon \left(\omega \right)=\epsilon_{r}^{\prime }\left(\omega \right)-j\epsilon_{r}^{\prime \prime }\left(\omega \right)\;$$

**Figure 3:**
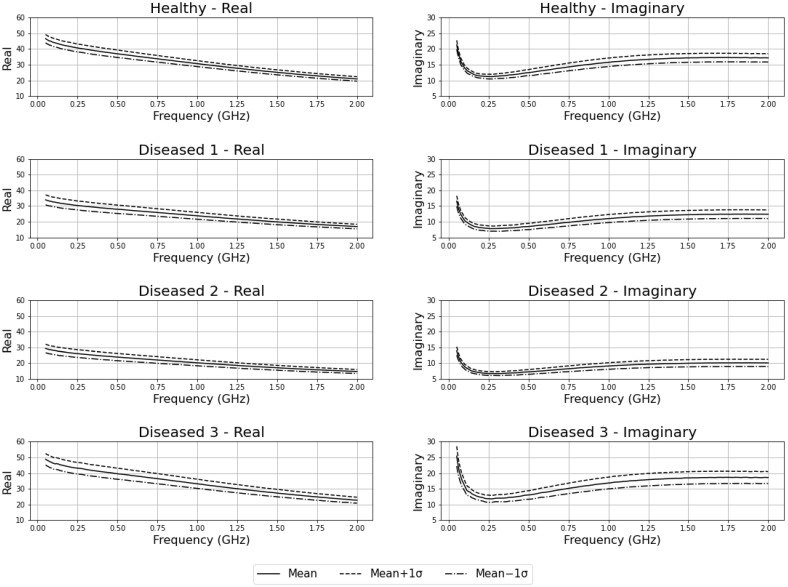
Measured complex permittivity for all tissue types individually.

**Figure 4:**
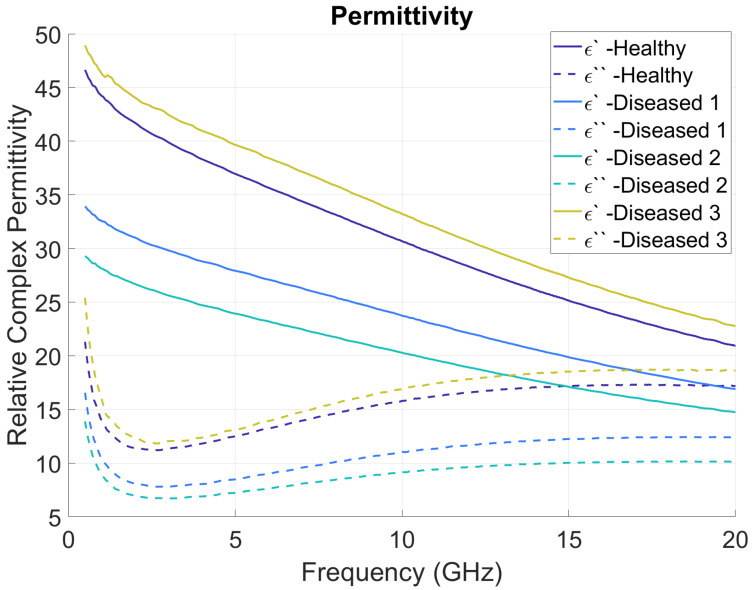
Average relative complex permittivity for healthy and diseased mice livers.

The measurement setup described in the previous section can collect both relative permittivity and loss factor of tissue at various frequencies. For this study, the measurements are saved across the 0.5 GHz to 20 GHz frequency range, with a step size of 39 MHz for each measurement. As a result of the frequency step size selected, each measurement output has 501 complex permittivity values (a total of 1002 values of dielectric constant and loss factor per measurement). Figure 3 shows the mean (solid line) and standard deviation (dotted lines) for real and imaginary components with different tissues. Figure 4 shows the average plots of all the measured data, grouped as healthy, diseased 1, diseased 2, and diseased 3. Solid lines show the real parts (${ɛ}^{\prime }$) of the measurements, and dotted lines show the imaginary parts (${ɛ}^{\prime \prime }$). The difference in the average of electrical characteristics between the four different tissue types can be understood by this plot. All the raw measured data plots for each sample are added in the Supplementary Material.

### 2.3. Classification Experiments

**Figure 5:**
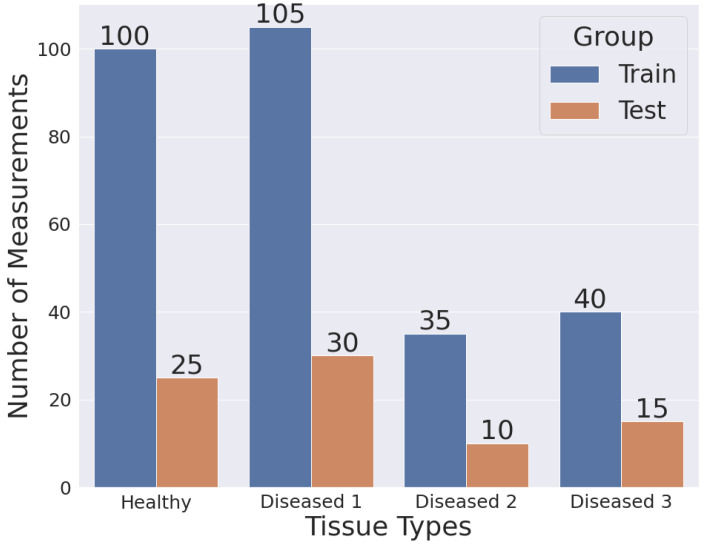
Train and test set count for each group.

We used the 501 points generated by real permittivity and 501 points generated by imaginary permittivity to create a 1002 feature vector for each measurement. From the 72 mouse tissue samples, we collected a total of 360 measurements for developing and testing our machine learning models. The measurements were divided into two independent datasets: training and testing. We divided the data such that measurements from a single sample were present in only one of the two sets. We did this to avoid the data leak of the same tissue sample in both training and testing sets using GroupShuffleSplit(), a function available in scikit-learn [28]. The function was also used to ensure that the number of samples in each set had similar class distribution. Figure 5 shows the number of measurements in the train and test sets.

We experimented with different machine learning classifiers, including logistic regression, k-nearest neighbors, random forest, and support vector machines, to perform a multi-class classification to discriminate between the healthy and three diseased tissue types. We used Python-based scikit-learn implementations of these machine learning algorithms to create the classification models [31].

We observed that our dataset was imbalanced with a relatively higher number of samples in healthy and diseased-1 classes. To address that, we used class weights during our model development. ‘Class_weights’ parameter of the scikit-learn implementation of the machine learning algorithms was set to ‘balanced’ for the experiments, except for the k-nearest neighbor algorithm. A ‘balanced’ class weight uses the values of samples per class to adjust weights inversely, proportional to class frequencies in the input data [32].

We performed a hyperparameter grid search for each classifier to obtain the optimal parameters to effectively discriminate between the four classes. The grid search for parameters was performed on the training data with 5-fold cross validation. The parameter search was optimized for best accuracy [31]. The optimized parameters were then used to train the model on the entire training dataset. The results are reported on the test dataset. The classifiers were evaluated based on precision, recall, F1 scores, and accuracy matrices.

## 3. Results

We performed classification using four different classifiers, including logistic regression, k-nearest neighbors, random forests, and support vector machines. Table 2 shows the classification performance for each classifier for the multi-class classification of the four types of liver tissues’ dielectric spectroscopy data on the hold-out test set. The table includes the weighted average precision, recall, and F1-score, along with the accuracy matrices for the multi-class classification.

Out of the four classifiers, SVM performed best with a 0.90 value for all performance matrices: precision, recall, F1-score, and accuracy. The logistic regression and random forest classifier performances were very similar, with accuracies 0.80, precisions 0.81, recalls 0.80, and f1 scores of 0.8 and 0.79 for the two classifiers, respectively. K-nearest neighbors did not perform as well as the other classifiers. This could be because of the class imbalance observed in the dataset. Logistic regression, random forest, and SVM classifiers could account for class balance using the ‘class_weight’ parameter. However, the k-nearest neighbors classifier does not have the ‘class_weight’ parameter in its implementation; hence, it did not account for the class imbalance in our dataset.

## 4. Discussion

This is the first study to the best of the authors’ knowledge that documents classification models to differentiate between NASH and fibrosis liver diseases. The ML models (logistic regression, k-nearest neighbors, random forest, and support vector machines) were optimized to maximize accuracy to differentiate variety of liver tissue types.

Conventional tissue diagnosis techniques require careful collection, processing, and assessment of samples by qualified physicians, where the subjectivity of the individual may have influence. However, applications in practically every field, including healthcare, artificial intelligence (AI), deep learning, and other machine-learning (ML) techniques, have made significant strides in recent years, as amplified by innovations in data science field [33]. ML is an essential component of the diagnostic workflow, improving treatment efficacy and outcomes for patients by reducing subjective errors and increasing diagnostic potency, while maintaining the reproducibility and accuracy of the results. Despite initial findings that are encouraging, the implementation of this technology requires big datasets to train complex neural networks, which can be difficult to achieve for the vast array of pathologies present and in the case of rare pathologies, even with data augmentation [34]. There is sufficient preliminary information from investigations on animal and human tissues to characterize their dielectric properties and detect any changes as they deviate from their normal state [4,35,36].

The data generated were split into two groups, as training and test sets, using balanced class weight. Four different machine learning models were used to analyze the data: logistic regression, k-neighbors classification, random forest classifier, and support vector classifier after optimization with grid searching. The support vector classifier achieved maximum accuracy at 90%. All the models performed equally, in terms of precision, recall, and F1 score. The findings imply that each tissue’s dielectric characteristics are distinct, and dielectric spectroscopy studies provide instantaneous and unique insights into tissue biopsy and excision, without inflicting any damage to the tissue architecture. This study shows how dielectric spectroscopy data may be combined with a machine learning model to rapidly identify aberrant tissues before they are processed in pathology labs. This rapid processing can aid in triaging patients and initiating empirical therapy if necessary. Once the dielectric characteristics of a considerable number of normal and pathological tissues are mapped, it can be employed as a vital intraoperative decision-making tool for assessing tumor-free margins and deciding the further extent of tissue dissection.

There are some limitations in this study. The sample size is limited to 72 mice liver with specific diseases. More research with bigger sample numbers and a varied range of diseases is required to validate the use of this technology and determine the dielectric signatures of all tissues for diagnostic or therapeutic purposes. The coaxial probe technique employed in this study to assess the dielectric co-efficient of tissues addresses many of the drawbacks of the existing methods. However, it assumes that the sample is homogeneous and in excellent contact with the probe; hence, air bubbles and uneven sample surfaces might lead to incorrect readings. A multitude of parameters, including probe design and materials (and hence impedance), precision of the probe production technique, calibration procedure (standards used), and measurement equipment capabilities, impact what may be measured (i.e., the VNA) [27].

The multi-class model offers huge promise in characterizing and differentiating a variety of liver diseases with reasonable accuracy from ex vivo experiments on relative permittivity. The future of this technology is the design and development of an AI-assisted, non-invasive biomedical sensor based on dielectric properties that can characterize various diseases in a patiently friendly manner in a clinical setting. This preliminary ex vivo study lays the foundation to build the database to optimize microwave biomedical sensor design and build more efficient machine learning algorithms to maximize diagnostic potential.

## 5. Conclusions

Four types of tissues are classified based on their electrical properties using ML techniques. The support vector machine model showed the best results and differentiated between different tissue types with 90% accuracy. Measuring electrical properties using a nondestructive method and non-ionizing microwave signal proved to be very effective. Combining the results with ML can produce fast and automated diagnosis possibilities for various diseases. Moreover, due to the penetration capabilities of microwave signals, this technology can be expanded to non-invasive diagnosis tools in the future.

## Figures and Tables

**Table 1:** Sample and measurement count.

Tissue Types	Samples	Measurements
Healthy	25	125
Diseased 1	27	135
Diseased 2	9	45
Diseased 3	11	55

**Table 2:** Classification results.

Classifier	Precision	Recall	F1-Score	Accuracy
Logistic Regression	0.81	0.80	0.80	0.80
K-Nearest Neighbors	0.76	0.75	0.74	0.75
Random Forest	0.81	0.80	0.79	0.80
Support Vector Machines	0.90	0.90	0.90	0.90

## Data Availability

The data plots are presented in the article/Supplementary Material, further inquiries can be directed to the corresponding author.

## Supplementary Materials

The following supporting information can be downloaded at: https://www.mdpi.com/article/10.3390/s22249919/s1, Figure S1: Measured Relative complex permittivity for healthy mice livers (25 samples); Figure S2: Measured Relative complex permittivity for Diseased 1 mice livers (27 samples); Figure S3: Measured Relative complex permittivity for Diseased 2 mice livers (9 samples); Figure S4: Measured Relative complex permittivity for Diseased 3 mice livers (11 samples).
