# Supplementary material for: Machine Learning-Based Classification of Abnormal Liver Tissues Using Relative Permittivity
Source: Sensors (Basel). 2022 Dec 16;22(24):9919. doi: 10.3390/s22249919 (PMC9781624; doi:10.3390/s22249919)
Supplement: Supplementary file 1 [file sensors-22-09919-s001.zip › sensors-2059335-supplementary.pdf]

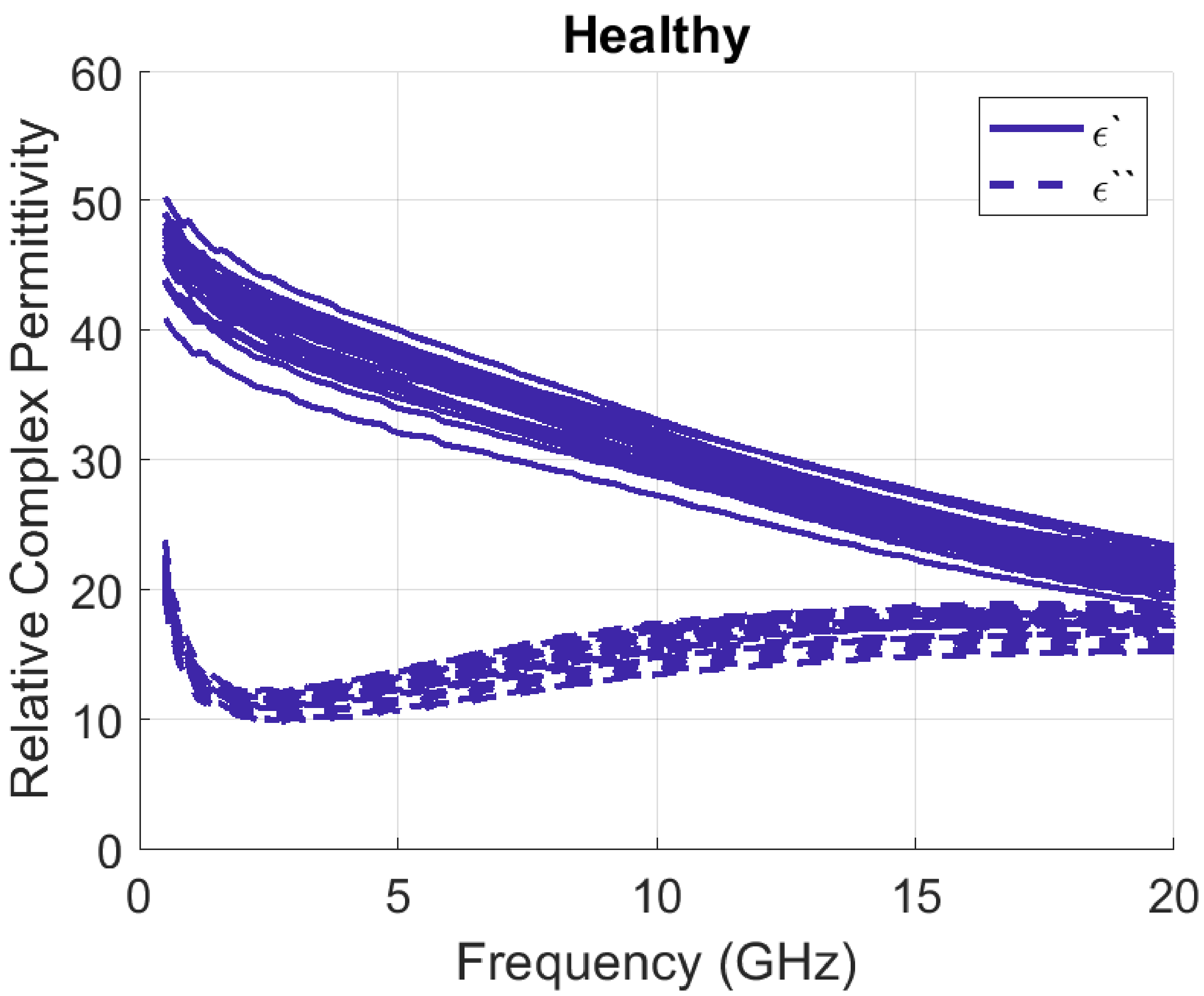

Figure S1: Measured Relative complex permittivity for healthy mice livers (25 samples)

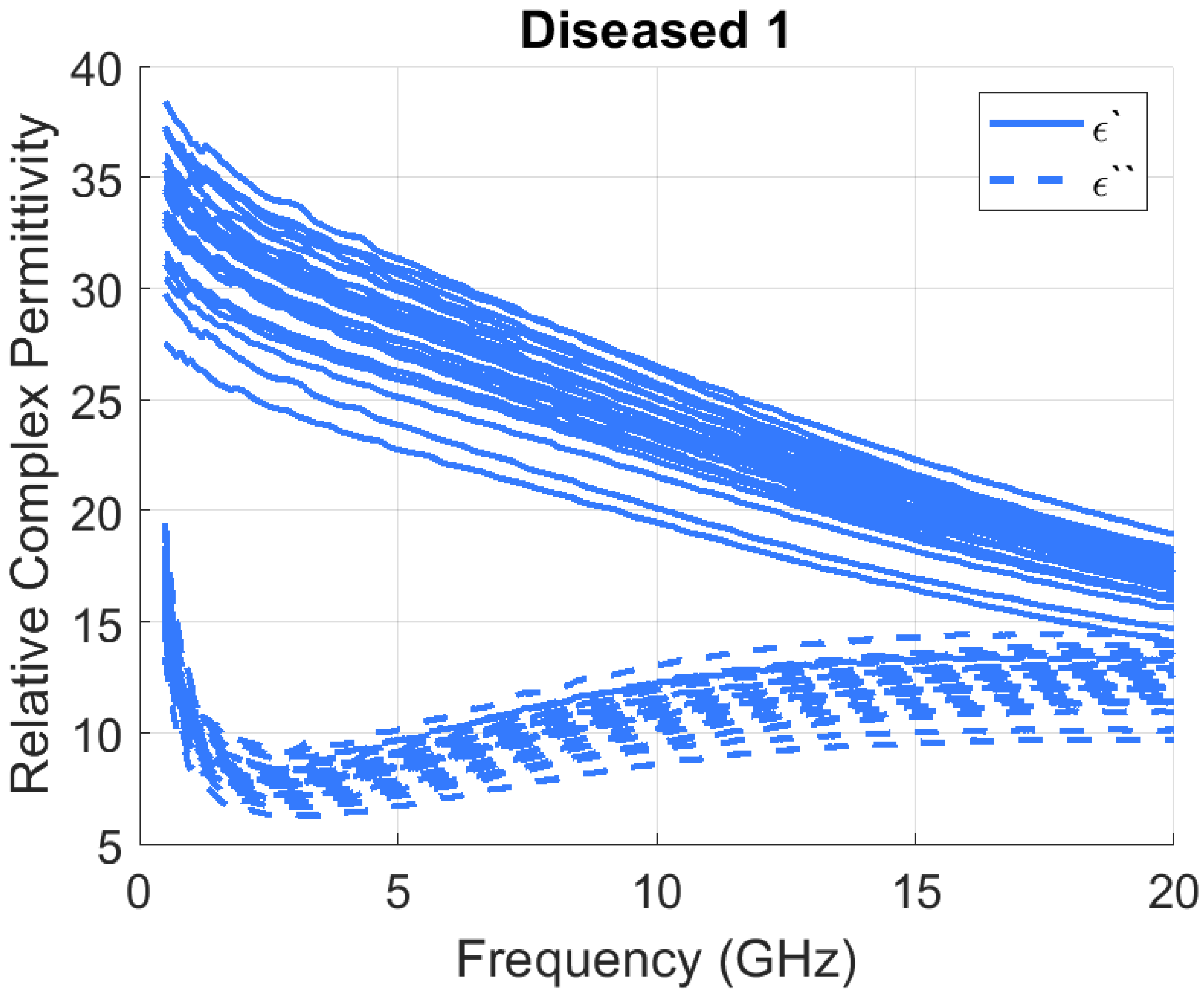

Figure S2: Measured Relative complex permittivity for Diseased 1 mice livers (27 samples)

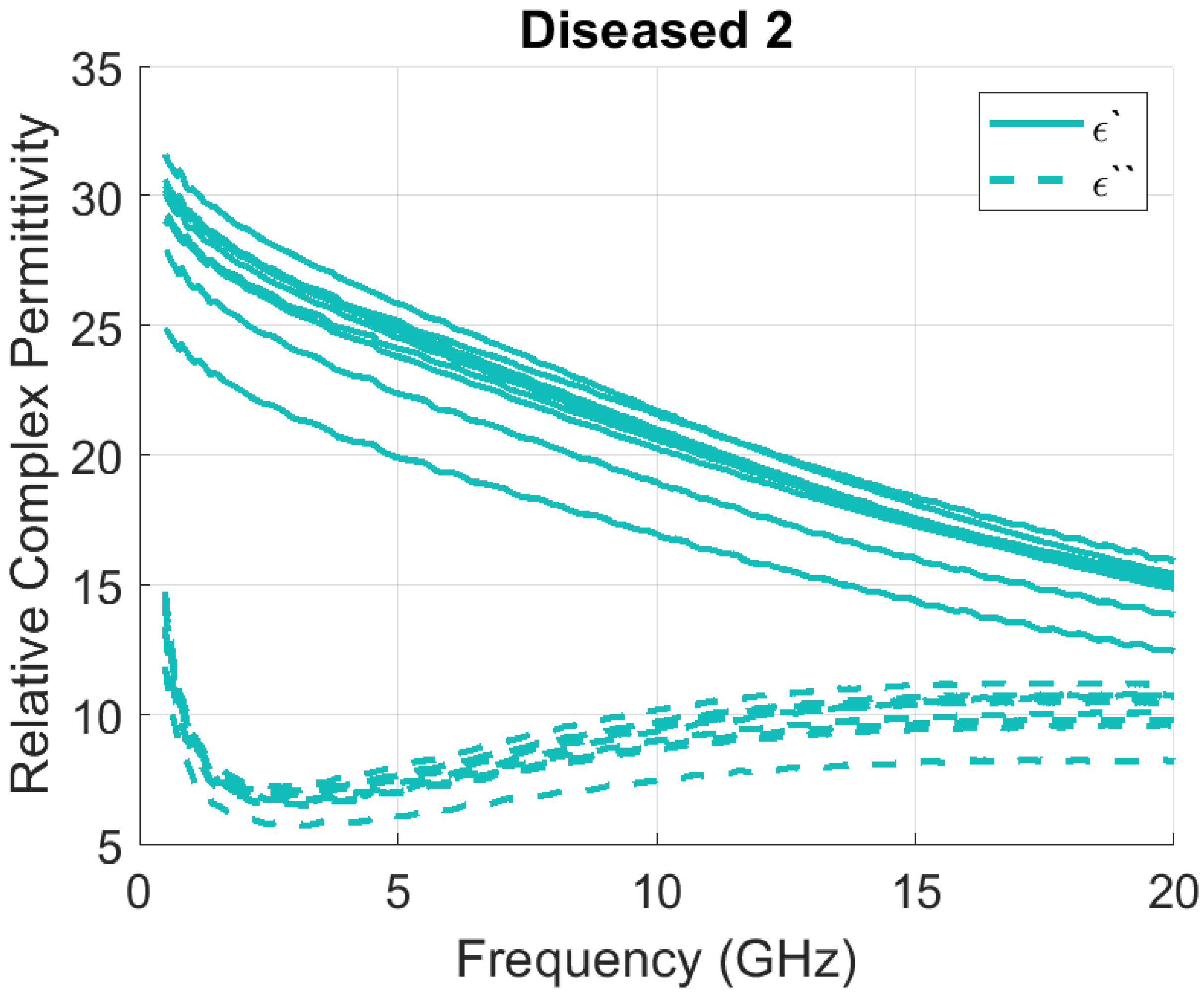

Figure S3: Measured Relative complex permittivity for Diseased 2 mice livers (9 samples)

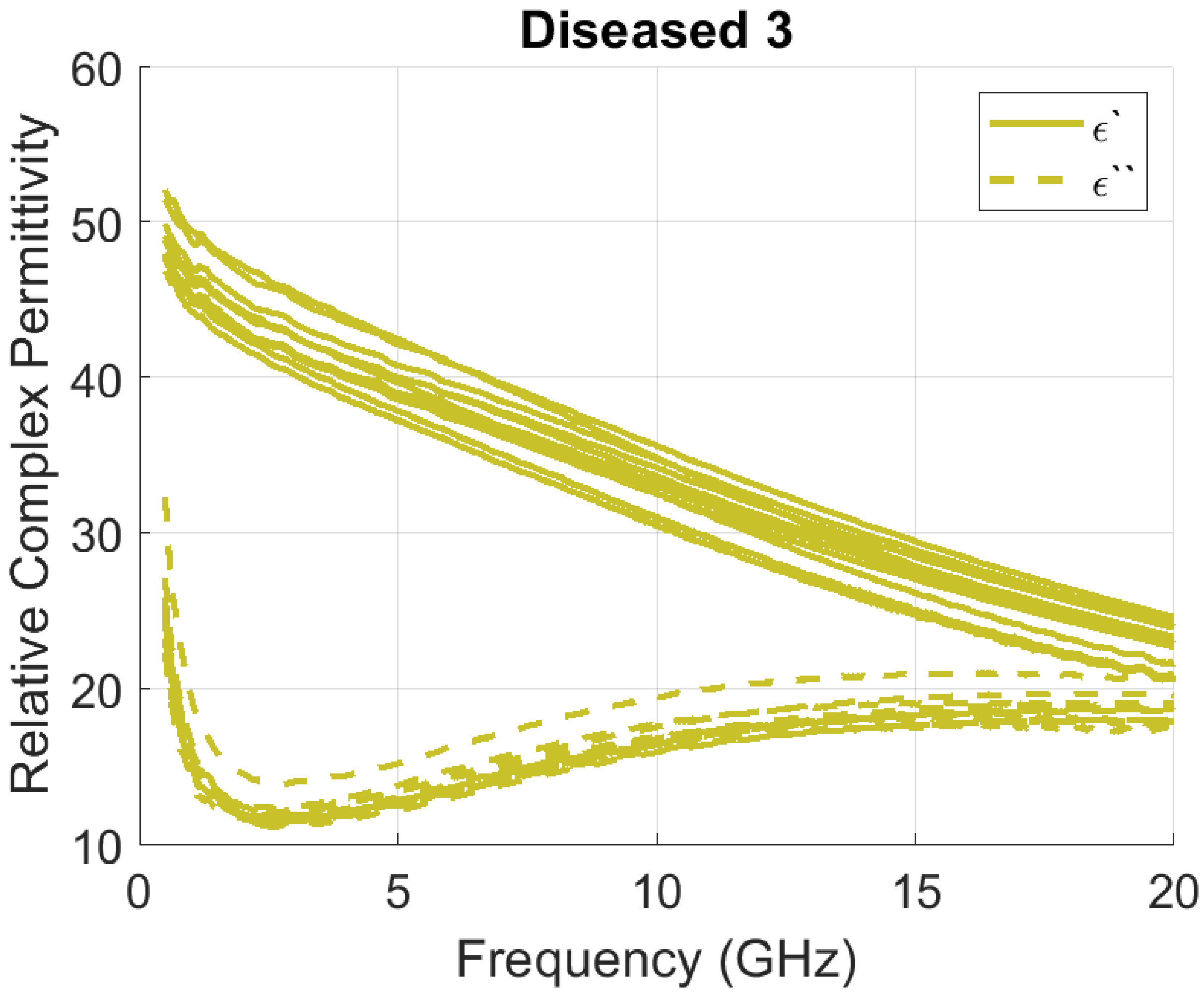

Figure S4: Measured Relative complex permittivity for Diseased 3 mice livers (11 samples)
